# The Bacterial Protein CNF1 as a Potential Therapeutic Strategy against Mitochondrial Diseases: A Pilot Study

**DOI:** 10.3390/ijms19071825

**Published:** 2018-06-21

**Authors:** Alessia Fabbri, Sara Travaglione, Zaira Maroccia, Marco Guidotti, Ciro Leonardo Pierri, Guido Primiano, Serenella Servidei, Stefano Loizzo, Carla Fiorentini

**Affiliations:** 1 Italian Center for Global Health, Istituto Superiore di Sanità, Viale Regina Elena, 299, 00161 Rome, Italy; alessia.fabbri@iss.it (A.F.); sara.travaglione@iss.it (S.T.); zaira.maroccia@iss.it (Z.M.); stefano.loizzo@iss.it (S.L.); 2 Department of Veterinary Public Health and Food Safety, Istituto Superiore di Sanità, Viale Regina Elena, 299, 00161 Rome, Italy; marco.guidotti@iss.it; 3 Department of Biosciences, Biotechnologies and Biopharmaceutics, University of Bari, via Orabona, 4, 70124 Bari, Italy; ciroleopierri@gmail.com or ciro.pierri@uniba.it; 4 Unità di Neurofisiopatologia, Area Neuroscienze, Università Cattolica del Sacro Cuore, Fondazione Policlinico Universitario Agostino Gemelli IRCCS, Largo Agostino Gemelli, 8, 00168 Rome, Italy; guido.primiano@gmail.com (G.P.); Serenella.Servidei@unicatt.it (S.S.)

**Keywords:** cytotoxic necrotizing factor 1, mitochondrial diseases, myoclonic epilepsy with ragged red fibers syndrome, actin cytoskeleton, adenosine triphosphate

## Abstract

The *Escherichia coli* protein toxin cytotoxic necrotizing factor 1 (CNF1), which acts on the Rho GTPases that are key regulators of the actin cytoskeleton, is emerging as a potential therapeutic tool against certain neurological diseases characterized by cellular energy homeostasis impairment. In this brief communication, we show explorative results on the toxin’s effect on fibroblasts derived from a patient affected by myoclonic epilepsy with ragged-red fibers (MERRF) that carries a mutation in the m.8344A>G gene of mitochondrial DNA. We found that, in the patient’s cells, besides rescuing the wild-type-like mitochondrial morphology, CNF1 administration is able to trigger a significant increase in cellular content of ATP and of the mitochondrial outer membrane marker Tom20. These results were accompanied by a profound F-actin reorganization in MERRF fibroblasts, which is a typical CNF1-induced effect on cell cytoskeleton. These results point at a possible role of the actin organization in preventing or limiting the cell damage due to mitochondrial impairment and at CNF1 treatment as a possible novel strategy against mitochondrial diseases still without cure.

## 1. Introduction

Mitochondrial diseases (MDs) are a heterogeneous group of genetic disorders that arise as a result of a dysfunction of mitochondrial oxidative phosphorylation, the main source of cellular adenosine triphosphate (ATP) that is under dual genetic control, with nuclear and mitochondrial DNA (mtDNA) that work in concert. These pathological conditions are characterized by a multitude of clinical features in different combinations, with isolated organ or more frequent multisystem involvement, and with varying rates of clinical progression, age of symptom onset, and overall clinical severity [1]. Tissues with high energetic demand, such as brain, skeletal, and cardiac muscle, are the most frequently affected. Typical features of MDs are muscle weakness, exercise intolerance, hearing loss, ataxia, incoordination, seizures, and cognitive deficits. One of the well-defined distinct mitochondrial syndromes is myoclonic epilepsy with ragged-red fibers (MERRF), which was described for the first time in 1980 [2] and is caused, in most cases, by mutations in *MT*-*TK* gene encoding tRNALys and with the A-to-G transition at nucleotide 8344 (m.8344A>G) detectable in over 80% of patients [3,4]. In particular, MERRF patients frequently show defective kinetics of cytochrome c oxidase (COX) [5,6] that are detectable also in fibroblast cultures from patients’ skin biopsies [7,8].

In spite of the progressive, and sometimes devastating disease course, no curative therapy options are available and even palliative therapeutic tools are inadequate [9]. Hence, there is the urgency to design new appropriate and feasible strategies. Different approaches have been proposed as innovative in their action at the molecular or cellular level. Their innovative potential, however, is still uncertain despite the promising results obtained in vitro [10]. In this context, several findings suggest the potential of mitochondrial dynamics-targeted therapeutics in diseases that involve abnormalities of the common final pathway of mitochondrial energy metabolism, including MDs [11,12,13,14].

CNF1 is an *Escherichia coli* protein toxin that specifically activates the family of Rho GTPases (including the Rho, Rac, and Cdc42 subfamilies) [15,16,17], thus modulating the organization of the actin cytoskeleton. It is known that changes in the actin cytoskeleton drive many aspects of cellular behavior, including contractility in skeletal muscle and synaptic plasticity in the central nervous system [18,19], and indeed CNF1 administration has effects on both skeletal muscle and neuronal cells [20,21]. From a molecular point of view, CNF1 effects are characterized by the activation of a complex signaling, mostly dependent on Rho GTPases modulation, which comprises the phosphoinositide 3-kinase (PI3K)/Akt/IκB kinase/NFκB pathway [22] and the cyclic adenosine monophosphate (cAMP)–protein kinase A (PKA) pathway [23]. The signaling pathways engaged by CNF1 are, obviously, at the basis of the different outcomes induced by the toxin. Indeed, the ability of CNF1 to boost the mitochondrial ATP production and to promote the elongation of mitochondria by phosphorylating the dynamin-related protein 1 (Drp1), a protein implicated in mitochondrial dynamics, has already been reported in vitro [23]. Moreover, the recruitment of Drp1 to mitochondria is eased by the actin cytoskeleton activity [24,25]. Specifically, intracerebroventricular injection of a single purified CNF1 dose induces a notable amelioration of the seizure phenotype in an absence seizure mouse model, with a significant augmentation in neuroplasticity markers, in mitochondrial ATP content, and in mitochondrial complex IV activity in mouse brain cortices [26]. These results are accompanied by an increase in phospho-Drp1 and a decrease in mitochondrial fission proteins (Drp1 and hFis) expressions. Also, when used as a therapeutic tool, CNF1 is able to rescue cognitive and respiratory chain activity impairment and to increase ATP synthesis in mouse models of Rett syndrome, a rare neurodevelopmental disorder [27,28,29], and ATP brain content in a mouse model of Alzheimer’s disease [30].

Therefore, starting from the above observations, our main hypothesis is that CNF1 may have a potential therapeutic value in those conditions sharing deficits in mitochondrial functions. To address this question, we have cultured fibroblasts from a skin biopsy derived from a MERRF patient carrying a specific mtDNA change (m.8344A>G mutation) and a partial defect of complex IV activity (Table 1). Our explorative results show that CNF1 is able to increase the energetic content, accompanied by a dramatic reorganization of the actin cytoskeleton, in human pathological primary cells.

## 2. Results

### 2.1. CNF1 Increases the ATP Content and Impacts Mitochondrial Morphology and Mass in Fibroblasts from MA19 Patient Biopsy

**Figure 1:**
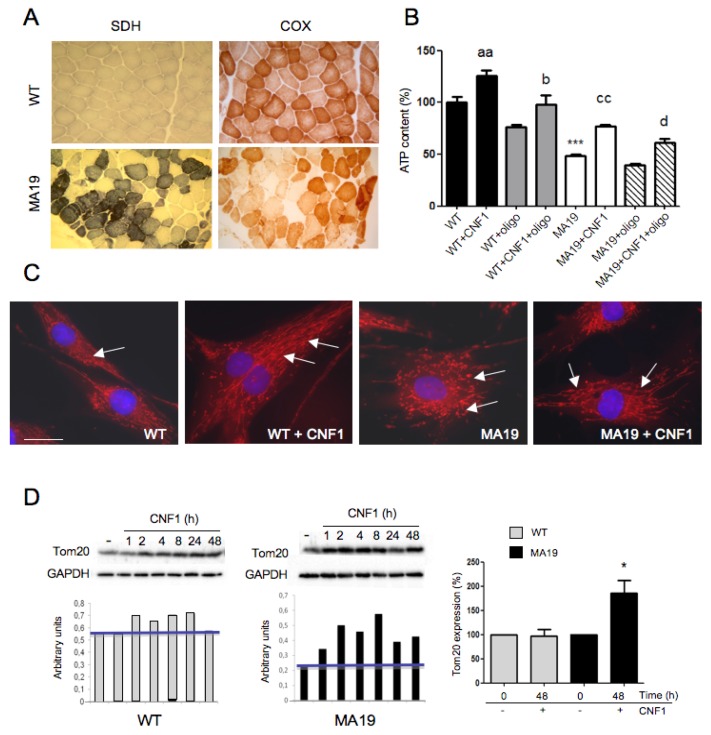
CNF1 effects ATP content and mitochondrial morphology and mass in fibroblasts from a patient carrying a mitochondrial disease. (**A**) Muscle pathology in a control patient (WT): the muscle looks normal without mitochondrial proliferation or COX-deficient fibers. Muscle pathology in the MA19 patient: SDH histochemistry shows mitochondrial proliferation with classic RBF, which appeared pale with the COX staining (magnification 20×); (**B**) Graph showing the relative increase of ATP content in WT and MA19 fibroblasts after 48 h of exposure to CNF1, either in the presence or absence of oligomycin added during the last 4 h of incubation. Data are expressed as percentages and represent mean ± standard error of the mean (SEM) from at least three independent experiments. *** *p* < 0.001 for WT versus MA19; aa *p* < 0.01 for WT versus WT + CNF1; b *p <* 0.05 for WT + oligo versus WT + CNF1 + oligo; cc *p* < 0.01 for MA19 versus MA19 + CNF1; d *p <* 0.05 for MA19 + oligo versus MA19 + CNF1 + oligo; (**C**) Fluorescence micrographs of WT and MA19 fibroblasts treated with CNF1 for 48 h and then stained with the mitochondrial dye Mitotracker (red) and with Hoechst 33258 (blue). Note that CNF1 treatment modifies the mitochondrial network in MA19 fibroblasts, rescuing the mitochondrial morphology of untreated WT fibroblasts. Bar = 10 µm; (**D**) Immunoblots showing representative Western blot experiments in whole cells lysates from control WT and MA19 fibroblasts. The amount of Tom20 is normalized as a function of GAPDH (bottom histograms). The histogram on the right shows, in percentage, the relative increase in Tom20 expression for both cell types (untreated WT and MA19 at time 0 = 100) following 48 h of toxin challenge, both normalized to their controls (treatment at time 0 = 100%). Data are represented as the means ± SEM from three independent experiments. * *p* < 0.05 for MA19 versus MA19 + CNF1.

For our pilot investigation, a patient harboring an m.8344A>G mutation was recruited (MA19120692, herein indicated as MA19). Symptoms were severe muscle weakness, respiratory insufficiency requiring ventilatory support, severe cardiomyopathy, deafness, peripheral neuropathy, and myoclonus. As a control, we used fibroblasts derived from a patient with non-mitochondrial myopathy and normal muscle morphology (herein indicated as WT) followed in Gemelli Hospital. Skin and muscle biopsies were collected for diagnostic purposes. In the MERRF patient, succinate dehydrogenase (SDH) staining showed myopathic changes and mitochondrial proliferation with classic RBF, which appeared pale with COX staining (Figure 1A), and biochemistry revealed a partial defect of respiratory chain complex IV activity as reported in Table 1.

To verify if fibroblasts cultured from skin biopsy of the MA19 patient present a decrease in ATP content with respect to fibroblasts cultured from a WT control patient, and to verify if CNF1 was able to rescue the bioenergetic defect in vitro, we treated fibroblasts with CNF1 for 48 h and analyzed the total ATP content by luminometric assay. To evaluate the possible mitochondrial activity contribution to the detected ATP levels, we also incubated with oligomycin, an inhibitor of ATP synthase, during the last 4 h of incubation. As shown in Figure 1B, WT fibroblasts present higher ATP levels with respect to MA19 cells, confirming the histochemistry and biochemical observations on muscle shown in Figure 1A and in Table 1. Also, a challenge with CNF1 for 48 h caused a significant increase in the ATP content in both MA19 and WT patient fibroblasts. The experimental groups treated with oligomycin showed a general decrement of ATP levels with a slight greater difference between the groups without the addition of oligomycin in the last 4 h of treatment, suggesting a mitochondrial contribution to the evaluated ATP production. We then asked whether CNF1 could somehow influence mitochondrial morphology, in both WT and MA19 fibroblasts, being CNF1 able to modulate mitochondrial shape [22,23]. To address this question, both fibroblast types were stained with the mitochondrion-selective dye Mitotracker after 48 h of challenge with CNF1. Micrographs (Figure 1C) evidenced that, while in untreated and in CNF1-challenged WT cells the mitochondrial network was well-organized in elongated and interconnected mitochondria, MA19 fibroblasts showed essentially fragmented and punctate mitochondria (Figure 1C). Interestingly, this last cell type seemed to benefit from the toxin action, showing mitochondria with a less fragmented phenotype similar to those observed in WT fibroblasts. Indeed, in CNF1-treated MA19 samples the percentage of cells with elongated mitochondria (calculated as reported in Materials and Methods) was very similar to that observed in control WT cells (WT: 30 ± 4%, WT + CNF1: 71 ± 7%, MA19: 5 ± 2%, MA19 + CNF1: 27 ± 5%). Western blot analysis showed that the expression level of Tom20, a component of the translocase of the outer mitochondrial membrane (Tom) which is the main mitochondrial entry gate for nuclear-encoded proteins [31], was transiently increased following treatment with CNF1 in both WT and MA19 fibroblasts with the maximum increment at 8 h. After 48 h of CNF1 treatment, whereas in WT the Tom20 expression returns to control level, in the MERRF patient’s fibroblasts the increase remains significant (*p <* 0.05) (Figure 1D). In this Figure, representative immunoblots are shown, while the graph reports the relative increase of Tom20 expression in each cell type at 48 h.

### 2.2. CNF1 Restores, in MA19 Fibroblasts, the Actin Stress Fibers’ Organization Typical of Control Cells

**Figure 2:**
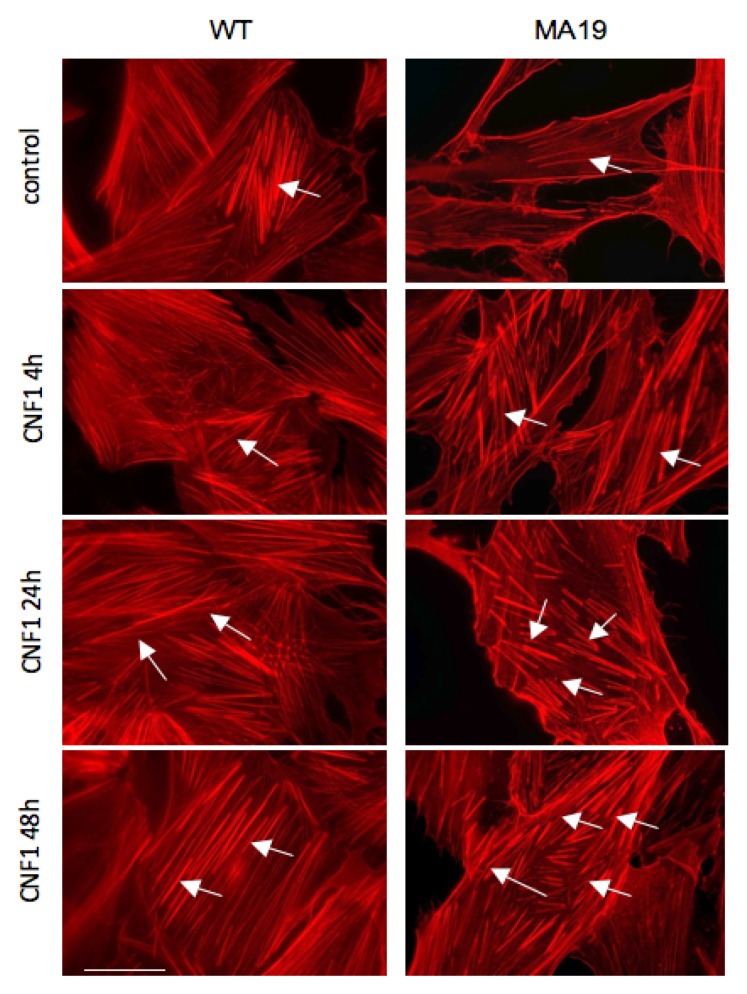
In the MA19 patient’s fibroblasts, CNF1 restores the actin stress fibers’ organization typical of WT cells. Fluorescence micrographs of WT fibroblasts (**left**) column and MA19 fibroblasts (**right**) column treated with CNF1 at the indicated time points. Note that the actin stress fibers’ organization appeared improved in WT fibroblasts with an apparent reinforcement of the actin fibers. The reorganization promoted by the toxin was more evident in pathological fibroblasts that showed an impressive restoration of the cytoskeletal phenotype typical of fibroblasts. Arrows show the stress fibers. Bar = 10 µm.

One of the main effects of CNF1 is the rearrangement of the cell cytoskeleton, particularly the actin network [32]. Being current knowledge that the actin cytoskeleton influences a plethora of cell functions, including mitochondrial shape and activity [33], we asked whether CNF1 could exert its influence on the actin network also in these human cells derived from two patients with a different diagnosis in terms of mitochondrial involvement. Staining with fluorescent phalloidin, which specifically links the polymerized filamentous actin (F-actin) [34], evidenced the capacity of CNF1 to improve the actin network in both cell types, inducing an augmentation and thickening of the stress fibers (Figure 2, indicated by arrows). The actin reorganization promoted by CNF1 was particularly evident in MA19 fibroblasts. Indeed, MA19 cells, whose actin was poorly organized with respect to WT fibroblasts, showed an impressive rescue of the cytoskeletal phenotype typical of WT fibroblasts following challenge with CNF1. This is in accordance with the toxin-induced effects observed on mitochondria and points at the actin cytoskeleton as a promising target for limiting or preventing the cell damage due to mitochondrial impairment.

## 3. Discussion

The present pilot study was designed to explore the therapeutic potential of the bacterial protein CNF1 versus mitochondrial diseases. The rationale for this investigation resides on the demonstrated ability of CNF1 to boost the mitochondrial ATP production in cells [23] and to normalize respiratory chain activity and ATP synthesis in pathological animal models [29]. Therefore, it was conceivable to hypothesize that CNF1 could be even more effective in diseases directly depending on alterations of the energetic metabolism, such as MDs. For this study, we decided to start by testing CNF1 on fibroblasts derived from skin biopsies of a patient carrying a mitochondrial myopathy, specifically MERRF (MA19), and on fibroblasts derived from skin biopsies of a patient carrying a non-mitochondrial myopathy (WT).

Our results show that the exposure to CNF1 triggers a significant increase in total cellular ATP content, and a transient increment in the cellular protein content of Tom20, in both WT and MA19 fibroblasts. Interestingly, the Tom20 expression remains significantly higher after 48 h of CNF1 treatment only in MA19 fibroblasts. Furthermore, an apparently beneficial effect on the pathological mitochondrial morphology is observed in MA19 challenged with CNF1. Therefore, we herein show mitochondrial elongation and increase in Tom20 expression that suggests an increment of the mitochondrial biomass and, interestingly, that both effects could be linked to the increment of ATP cell content triggered by the toxin. In fact, in this study on fibroblasts, although we are aware that the total ATP content is not a measure of the mitochondrial ATP, the results obtained using oligomycin led us to speculate that the increased ATP content involves the mitochondrial activity, hence reinforcing our hypothesis. Interestingly, we have also observed that the above-described changes caused by CNF1 in MA19 fibroblasts were accompanied by a profound F-actin reorganization. In fact, whereas the actin cytoskeleton of WT fibroblasts showed the typical fibroblast-like architecture characterized by well-organized stress fibers, a feature that CNF1 further improved, the actin stress fibers of MA19 fibroblasts appeared poorly organized. In this last case, the action of CNF1 was particularly impressive, restoring the typical phenotype of control fibroblasts. Actually, CNF1 is a well-known modulator of Rho, Rac, and Cdc42 GTPases, molecular switches that directly or indirectly affect the local actin assembly or disassembly [35]. Interesting, a growing body of evidence suggests that the cytoskeleton regulates not only mitochondrial movement but also mitochondrial morphology and functionality [33] or, in general, mitochondrial network homeostasis [36]. In this context, it appears reasonable to speculate that the actin reorganization, observed in MA19 fibroblasts challenged with CNF1, may play a pivotal role in the energetic content increment. Hence, our hypothesis is that the actin cytoskeleton, which appears dysregulated in fibroblasts from a patient carrying a mitochondrial mutation, may act as a pharmacological target against those diseases that present energy production impairment.

Although a single case cannot allow a general conclusion and further experiments are mandatory to clarify the CNF1 effect on mitochondria, this first exploration adds evidence to the role of CNF1 as an energetic metabolism regulator [37], thus paving the way towards the use of the toxin as a potential therapeutic tool against diseases involving mitochondrial defects. Further studies are obviously required to confirm our preliminary results and to clarify the link between the actin reorganization and the energy increment promoted by the toxin. Also, the evaluation of all energetic sources that may contribute to the cell energetic increase triggered by CNF1 will be the focus of future investigations.

## 4. Materials and Methods

### 4.1. Patients Recruitment and Histochemical Characterization

A patient harboring an m.8344A>G mutation and followed at the Neurophysiopathology Unit of Gemelli Hospital was investigated. Muscle and skin biopsies were collected for diagnostic purposes after written informed consent. Cryosections were analyzed using standard procedures [38] and respiratory chain enzymes activities were measured in muscle as previously described [39]. The MERRF patient started complaining of exercise intolerance from 20 years of age. Currently, he has severe muscle weakness and cardiomyopathy with an implantable cardioverter-defibrillator, deafness, peripheral neuropathy, myoclonus, and respiratory insufficiency requiring ventilatory support. Laboratory data are summarized in Table 1. Control fibroblasts (WT fibroblasts) were derived from a patient that underwent muscle and skin biopsies for a persistent hyperCKemia in which, however, morphology, hystochemistry, and biochemistry showed no abnormalities.

All subjects gave their informed consent for inclusion before they participated in the study. The study was conducted in accordance with the Declaration of Helsinki, and the protocol was approved by the Ethics Committee of Istituto Superiore di Sanità, Rome (Project identification code: Prot. PRE 123/18, 15 February 2018).

### 4.2. Fibroblasts and Treatments

Fibroblasts were isolated from skin punch biopsies and histochemically characterized according to the Vangipuram et al. protocol [40]. Fibroblasts were grown in Dulbecco’s Modified Eagle’s medium supplemented with 10% fetal bovine serum (Gibco, Carlsbad, CA, USA), penicillin (100 U/mL), and streptomycin (100 μg/mL, Lonza, Basel, Switzerland). All experiments were performed on cells with similar passage numbers (8–20) to avoid artifacts due to senescence, which are known to occur at passage numbers greater than 30. Once they reached confluence, cells were exposed for 4 h, 24 h, and 48 h to 10^−10^ M CNF1.

To inhibit mitochondrial ATP synthase activity, 4 h before the end of CNF1 treatment, 10 μg/mL oligomycin (Sigma-Aldrich; Saint Louis, MI, USA) were added to the culture medium.

### 4.3. CNF1 Preparation and Purification

CNF1 was obtained from the 392 ISS strain (provided by Vincenzo Falbo, Istituto Superiore di Sanità, Rome, Italy) and purified essentially as previously described [41] with a few modifications in the procedure.

### 4.4. Fluorescence Microscopy

Control and CNF1-treated fibroblasts, seeded on glass coverslips, were fixed in 4% paraformaldehyde in phosphate-buffered saline (PBS) and permeabilized with Triton X-100 (0.2%, Sigma-Aldrich, St. Louis, MO, USA). For F-actin detection, cells were stained with FITC (fluoresceine isothyocianate)-phalloidin (Sigma-Aldrich; working dilution 0.5 µg/ mL in PBS) for 30 min at 37 °C. For mitochondrial staining, cells were incubated with 1 µM Mitotracker Red CMXRos (Invitrogen, Carlsbad, CA, USA) for 1 h before fixation. Nuclei were stained with Hoechst 33258 (Sigma-Aldrich, St. Louis, MO, USA). Finally, following extensive washes, samples were mounted on glass coverslips and observed with an Olympus BX51 fluorescence microscope (Tokyo, Japan).

The percentage of cells bearing elongated mitochondria was calculated on a total of at least 200 cells observed from different regions of the glass coverslips. Only cells containing more than 20% elongated mitochondria were considered.

### 4.5. Protein Extraction and Western Blot

Cells were lysed in RIPA buffer (50 mM Tris-HCl, pH 7.4, 150 mM NaCl, 1% NP-40, 2 mM EDTA plus 10 µg/ mL aprotinin, 10 µg/ mL leupeptin, 1 mM PMSF, 1 mM Na_2_VO_4_) at +4 °C according to standard procedures. Twenty-five total protein extracts were resolved on 12% SDS/PAGE and electrically transferred onto poly(vinylidene difluoride) membranes (Bio-Rad Laboratories, Hercules, CA, USA). Membranes were blocked with TBS-T (20 mM Tris/HCl, pH 7.4, 150 mM NaCl, 0.02% Tween-20) containing 5% skimmed milk (Bio-Rad Laboratories) for 1 h at room temperature and then incubated overnight at 4 °C with the following primary antibodies diluted in TBS-T containing 5% milk: rabbit polyclonal anti-Tom20 (1:500; Santa Cruz Biotechnology, Dallas, TX, USA) and mouse monoclonal anti-GAPDH (1:5000; Sigma-Aldrich). After thorough washing in TBS-T, immunocomplexes were revealed with horseradish-peroxidase-conjugated species-specific secondary antibodies (Jackson Laboratory, Bar Harbor, ME, USA) followed by an enhanced chemiluminescence reaction (Millipore Corporation, Darmstadt, Germany). Reactive bands were detected by the ChemiDoc MP system (Bio-Rad Laboratories) and signal quantification was performed using the IMAGE LAB software 5.0 (Bio-Rad Laboratories).

### 4.6. Measurement of ATP Content

The content of cellular ATP was assayed luminometrically using the ATPLITE 1 STEP (PerkinElmer, Waltham, MA, USA) according to the procedure recommended in the manufacturer’s instructions and using 1 × 10^4^ cells. Briefly, fibroblasts were homogenized in 50 µL of lysis buffer and mixed for 10 min. Forty microliters of substrate solution (Luciferase/Luciferin) was added to each sample. The luminescence was measured using a luminescence plate reader (Victor3-V, PerkinElmer). The ATP concentration was normalized to the total cell protein content evaluated by Bradford protein assay (Bio-Rad Laboratories).

### 4.7. Statistical Analysis

All experiments were done at least in triplicate. Data was presented as mean ± standard error of the mean (SEM). An independent samples *t*-test or one-way ANOVA, followed by Dunnet’s post-hoc test, was used. Correlation was considered as significant when the *p* value was <0.05.

## Figures and Tables

**Table 1:** Clinical, morphological, molecular and biochemical data on the selected myoclonic epilepsy with ragged-red fibers (MERRF) patient. Severity of myopathological features: absent (−) to severe (++++).

Description	MERRF
age at biopsy (years)	50
ragged-blue fibers (RBF)	+++
cytochrome c oxidase (COX) negative RBF	+
% m.8344A>G mtDNA mutation	muscle: 78; blood: 65
% muscle COX activity (normalized for citrate synthase)	60

## Abbreviations

CNF1	cytotoxic necrotizing factor 1
MERRF	myoclonic epilepsy with ragged-red fibers
MDs	mitochondrial diseases
RBF	ragged-blue fibers
mtDNA	mitochondrial DNA
Drp1	dynamin-related protein 1
SDH	succinate dehydrogenase
COX	cytochrome c oxidase
Tom20	translocase of the outer mitochondrial membrane
F-actin	filamentous actin
